# An optimized novel lightweight block cipher for image encryption

**DOI:** 10.1038/s41598-025-19951-2

**Published:** 2025-10-15

**Authors:** R. Mohanapriya, Kumar V. Nithish

**Affiliations:** School of Electronics Engineering, Vellore Institute of Technology, Vellore, Tamilnadu 632014 India

**Keywords:** Lightweight cryptography, ARX cipher, Cryptanalysis, Image encryption, Key schedule, FPGA implementation, Engineering, Electrical and electronic engineering

## Abstract

In the era of pervasive multimedia communication, image data has become a dominant form of information exchange across embedded, mobile, and IoT platforms. This surge in visual data transmission introduces critical challenges related to confidentiality, authenticity, and tamper resistance particularly in resource-constrained environments where conventional cryptographic solutions may prove computationally intensive. To address these challenges, lightweight cryptographic algorithms tailored for image protection are essential, balancing rigorous security requirements with efficient hardware and software implementation. This paper proposes a novel lightweight block cipher optimized for image encryption, employing a multi-stage internal Addition-Rotation-XOR (ARX) structure within each round to enhance confusion and diffusion. The cipher operates on 64-bit plaintext blocks with a 64-bit master key and utilizes a customized key schedule mechanism that generates five distinct subkeys per round through bit-swapping, modular addition, and XOR operations. The cryptographic properties of the proposed cipher were evaluated using the NIST SP 800-22 statistical test suite, confirming high key randomness. Further analysis demonstrated robust security with a 50% average avalanche effect, a maximum differential probability of approximately $\lesssim 2^{-32}$, and a maximum linear bias below $\lesssim 2^{-8}$. The cipher achieves strong resistance to differential and linear cryptanalysis within five rounds, offering an optimal balance between security and efficiency. Comprehensive statistical analysis using various input images are analyzed and demonstrate the cipher’s robustness in securing visual data. The encryption algorithm was further implemented on an Artix-7 FPGA, and synthesis results confirmed its suitability for resource constrained environments. The results indicate that the proposed cipher offers a secure and efficient solution to modern image security challenges.

## Introduction

In the current digital era, the rapid transmission and widespread availability of images have become integral aspects of daily life. Consequently, ensuring the security and integrity of these images has become increasingly critical^1,2^. To comply with regulatory requirements, image data must be protected against unauthorized access. One of the primary concerns in the transmission and storage of images is ensuring secure communication, which is essential for safeguarding image data during transmission^3^. Image encryption is essential for ensuring the security of multimedia applications in digital distribution networks^4^. Traditionally, classical cryptographic techniques like watermarking^5^, stenography^6^, and chaotic algorithms^7^ have been employed to ensure the security of text, image, audio, and video data. Among these, the most widely used methods for encrypting images transmission conventional ciphers, including the Advanced Encryption Standard (AES)^8^, Twofish^9^, and Rivest–Shamir–Adleman (RSA)^10^. As conventional encryption algorithms are unsuitable for resource-limited environments due to their high computational complexity, several lightweight encryption algorithms have been studied in recent years for use in resource-constrained devices such as IoT devices, RFID tags, and sensors^11–16^. Additionally, the NIST launched the lightweight cryptography project over a decade ago to address the need for secure and efficient cryptographic solutions for constrained environments. In parallel, the international standard ISO/IEC 29192 was developed by ISO/IEC JTC 1/SC 27 to provide guidelines for lightweight cryptographic algorithms. Due to their minimal computational complexity, lightweight cryptographic architectures are well-suited for integration into resource-constrained technologies^17^.

In general, the architecture of lightweight block ciphers are classified into three main types: Substitution-Permutation Network (SPN), Feistel, and ARX-based designs. SPN-based ciphers, which typically require more hardware resources due to the implementation of substitution boxes (S-boxes), include RECTANGLE^18^, PRESENT^19^, and GIFT^20^. Similarly, Feistel-based algorithms include HIGHT^21^,Camellia^22^, DESXL^23^ and Piccolo^24^. These ciphers are designed to achieve a high degree of confusion and diffusion through a larger number of encryption rounds operations. Likewise, the ARX based structures such as CHASKEY^25^, LEA^26^, CHAM^27^, SPECK^28^, Salsa20^29^, SPARX^30^, and ChaCha^31^ are known for their low power consumption and simpler hardware implementation compared to other cryptographic structures.

In recent years, ARX based encryption techniques based on simple arithmetic operations have gained significant attention due to their inherent simplicity, computational efficiency, and strong diffusion properties. These characteristics make ARX ciphers highly suitable for lightweight and hardware-constrained environments. However, existing ARX-based designs often face trade-offs between security strength, randomness, and hardware efficiency. One of the most critical aspects affecting these trade-offs is the key schedule mechanism, which must ensure high unpredictability and resistance to cryptanalytic attacks. Achieving strong randomness in sub round key schedule is therefore essential to enhance the overall security and resilience of the cipher. Guard Kanda et al.^32^ present a low-area, high-throughput ChaCha20 stream cipher architecture designed for secure hardware communication. The implementation leverages pipelining and parallel processing techniques to enhance frequency performance and area efficiency. Johannes Pfau et al.^33^ propose an efficient hardware design for the ChaCha cipher, utilizing pipelining, block memory, and register-based techniques. Their fully pipelined architecture is demonstrated to outperform multiple processing cores in terms of throughput for high-performance applications. Sugier Jarosław^34^ explores the FPGA implementation of the 256-bit Salsa20 stream cipher, which is renowned for its security and speed. The study evaluates the performance of the loop-unrolled and pipelined architectures of Salsa20 on hardware platforms. Jun-Hoe Phoon et al.^35^ present a FPGA implementation of lightweight LED and SIMECK ciphers on the Xilinx Artix-7, achieving high performance and efficiency. In the LED cipher, a lookup table replaces multipliers in the mixColumns operation, reducing resource usage. Meanwhile, SIMECK features the most compact parallel architecture, making it ideal for resource-limited applications. Youssef et al.^36^ introduce a new pseudo-chaotic random number generator for the SPECK cipher and implement it on FPGA. The design meets the security level requirements for communication among IoT devices.

Zeesha Mishra et al.^37^ introduce a high-speed, low-area unified LEA architecture for resource-constrained devices, supporting 128, 192, and 256-bit keys through ARX operations. The pipelined design enhances the operating frequency with modified key schedule method which optimize hardware resources. Gaurav Uttam et al.^38^ present an efficient hardware implementation of the Improved-LEA block cipher for IoT devices, utilizing pipeline-based and round-based techniques. The pipelined architecture for 128-bit and 192-bit keys enhances throughput, while the round-based design optimizes area efficiency. Kiran Kumar et al.^39^ present the BRIGHT and SIMON (BRISI) lightweight algorithms designed for low-resource-constrained devices. The BRISI lightweight block cipher, based on ARX operations, is evaluated against standard hardware and security performance metrics using 32-bit and 64-bit keys. Asmita Poojary et al.^40^ present a modified-BRISI (MBRISI) cipher, operates on 32-bit plaintext with an optimized 64-bit key schedule module. Nagesh et al.^41^ introduce the HIBRI cipher, which combines the HIGHT and BRIGHT ciphers aims to provide a more robust and flexible encryption solution that retains the lightweight nature of both algorithms while enhancing security. Its implementation in both software and hardware ensures adaptability across different platforms, making it suitable for modern resource-constrained systems. Kiran Kumar et al.^42^ introduce ARX/MRX encryption schemes for IoT security, utilizing Addition-Modulo, Multiplication-Modulo, Rotation, and XOR operations. The designs are implemented with reversible logic and Vedic multiplier and is optimized for low-resource devices. To achieve high performance, Raja et al.^43^ proposed the SIMECK cipher on an FPGA hardware platform, employing various optimization techniques such as loop unrolling, inner pipelining, and outer pipelining. Wen Chen et al.^44^ introduced DABC, a dynamic ARX-based lightweight block cipher featuring a dynamic permutation layer. Both ASIC and FPGA implementations demonstrate its efficient hardware resource utilization. Xing Zhang et al.^45^ proposed the GFRX algorithm, which combines a generalized Feistel structure with ARX operations and diverse nonlinear components, enhancing diffusion and enabling flexible serialization tailored for resource-constrained hardware. This work introduces a novel lightweight ARX cipher that addresses the dual challenges of hardware efficiency and security strength. By incorporating an multiple subkeys generation key scheduling mechanism based on modular addition, XOR, and bit-swapping operations the proposed design ensures high randomness across the several rounds. Unlike conventional ARX based ciphers, the proposed architecture utilize multi-stage, multi-key approach significantly improves security compared to traditional ARX designs with repetitive structures.

The rest of the paper is organized as follows: Section 2 presents the architecture of the proposed lightweight ARX cipher along with the novel key scheduling mechanism. Section 3 provides a detailed security analysis of the proposed cipher. Section 4 discusses the hardware implementation and performance evaluation on an FPGA platform. In Section 5, comprehensive statistical analysis on encrypted images is conducted to validate the cipher’s effectiveness. Finally, Section 6 concludes the paper and outlines directions for future work.

## Design of the proposed lightweight ARX cipher

This section presents the detailed design of the proposed lightweight ARX cipher, emphasizing both the novel key schedule mechanism and the overall encryption architecture. The design aims to achieve a balanced trade-off between security strength, randomness, and hardware efficiency.

### Encryption algorithm

**Fig. 1:**
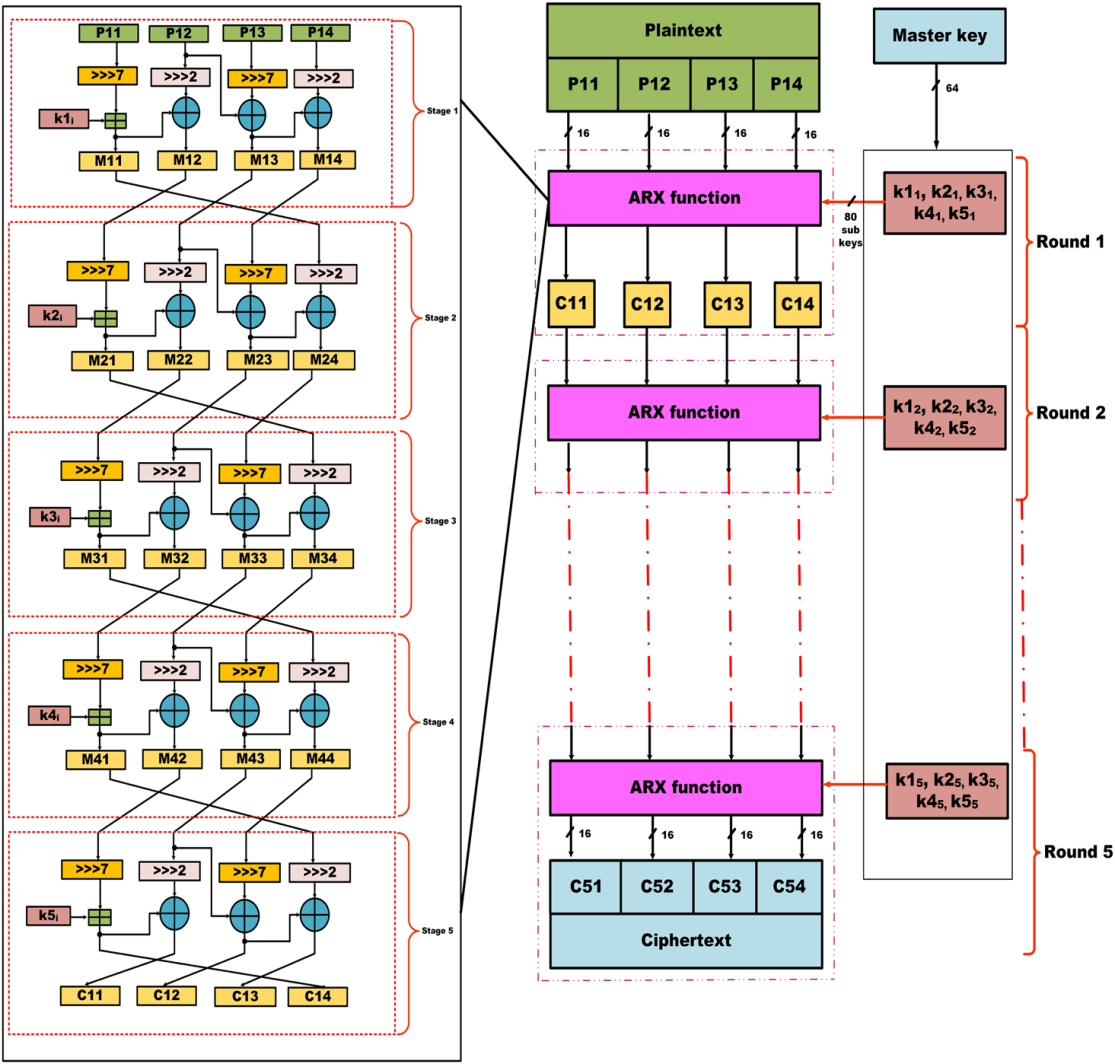
Block diagram of the proposed lightweight ARX cipher showing the encryption structure for five rounds, incorporating multi-stage operations.

The proposed cipher architecture is based on the ARX structure designed to efficiently perform encryption on 64-bit plaintext using a 64-bit master key. Each encryption round consists of five sequential stages, incorporating operations such as modular addition and bitwise XOR, with each stage utilizing a distinct sub-key derived from the novel key scheduling mechanism. This multi-stage, multi-key approach significantly improves security compared to other ARX designs with repetitive structures. To ensure strong diffusion and non-linearity in the ciphertext, the encryption process is to be carried out for five rounds of operation. Fig. 1 illustrates the block diagram of the proposed lightweight ARX cipher showing the encryption structure for five rounds, incorporating multi-stage operations. Table 1 presents the corresponding logical operation for the list of symbols presented in this paper for more clarity.

**Table 1:** List of symbols.

Symbols	Operations
$>>>$ 7	Circular right shift by 7
$>>>$ 2	Circular right shift by 2
$<<<$ 7	Circular left shift by 7
$<<<$ 2	Circular left shift by 2
$\oplus$	XOR operation
$\boxplus$	Addition modulo operation
$\boxminus$	Subtraction modulo operation

Let *i* represents the $i^{th}$ encryption round, where *i* = 1, 2, .... 5 for the proposed cipher. In the first round of operation, the 64-bit plaintext input is divided into four equal 16-bit segments from MSB to LSB denoted as *P*11, *P*12, *P*13, and *P*14, respectively. Then, *P*11 undergoes a circular right shift by seven bit positions and the resulting value is modulo added with the subkey $k1_{i}$ to produce the first quarter output, denoted as *M*11. Next, *P*12 is circularly right shifted by two bit positions and XOR-ed with *M*11 to produce the second quarter output, denoted as *M*12. Similarly, *P*13 is circularly right-shifted by seven bit positions and the result is XOR-ed with *M*12 produce the third quarter output, denoted as *M*13. Finally, *P*14 is circularly right-shifted by two bit positions and XOR-ed with *M*13 yielding the fourth quarter output, denoted as *M*14. After that, the generated quarter outputs are rearranged in the specific order *M*12, *M*13, *M*14 and *M*11 to form the first-stage 64-bit intermediate encrypted output. This reordered output is then forwarded to the subsequent stages, where it undergoes similar operations using the corresponding subkeys i.e $k2_{i}$, $k3_{i}$, $k4_{i}$, $k5_{i}$ to ultimately generate the ciphertext for a single round of encryption. Likewise, the remaining four rounds of the encryption process are executed in a similar methodology. For each subsequent round, the input is derived from the output of the previous round, which is reordered according to a predefined pattern i.e., *M*12, *M*13, *M*14, and *M*11, respectively. This reordering enhances diffusion and key dependence across rounds. After the completion of all five rounds, the final outputs are concatenated to generate the ciphertext.

### Pseudocode for encryption process

Input: 64-bit plaintext split into four 16-bit segments *P*11, *P*12, *P*13 and *P*14. Five distinct 16-bit subkeys ($ks_{i}$) correspond to $s^{th}$ stage in the $i^{th}$ encryption round.

Output : 64-bit ciphertext

Step 1 : For i = 1 to 5 do; Loop over encryption rounds

Step 2 : For s= 1 to 5 do; Loop over stages within a single round

Step 3: *P*11 is circularly right shifted by seven times.

Step 4: Compute modulo addition $\boxplus$ with subkey $k1_{i}$ to produce the first quarter output: *M*11 = $P11 \ggg 7$ $\boxplus$ $k1_{i}$;

Step 5: *P*12 is circularly right shifted by two times.

Step 6 : Perform the bitwise XOR operation with *M*11 to produce the second quarter output: *M*12 = $P12 \ggg 2$ $\oplus$ *M*11;

Step 7: Circular right shift *P*13 by seven times and bitwise XORed with *P*12 to produce the third quarter output: *M*13 = $P13 \ggg 7$ $\oplus$ *P*12;

Step 8: Circular right shift *P*14 by two times and bitwise XORed with *M*13 to produce the fourth quarter output: *M*14 = $P14 \ggg 2$ $\oplus$ *M*13.

Step 9: Rearrange the quarter outputs as in the order *M*12, *M*13, *M*14, *M*11 and pass this as the input for the next stage

Step 10: Use the corresponding stage subkeys $k2_{i}$, $k3_{i}$, $k4_{i}$, and $k5_{i}$, respectively for the stages s = 2 to 5.

end

Step 11: Repeat from step 1 to 10 until *i* = 5 and concatenate the final outputs *C*51, *C*52, *C*53, *C*54 to get the 64-bit ciphertext.

end

Similarly, the decryption process of the proposed lightweight ARX cipher is performed by reversing the operations used during encryption. It involves processing the 64-bit ciphertext over five rounds in reverse order from round i = 5 to 1. In each round, the inverse operations of encryption are applied across five stages, using the corresponding subkeys in reverse sequence i.e., from $k5_{i}$ to $k1_{i}$. The inverse operations consist of the bitwise-XOR $\oplus$, since XOR is its own inverse, circular left shifts (to reverse the circular right shifts), and modulo subtraction $\boxminus$ (to reverse modular addition $\boxplus$ ). This reversed operation ensures complete reversibility of the encryption process while maintaining the cipher’s lightweight and secure properties.

### Architecture of the novel key schedule mechanism

**Fig. 2:**
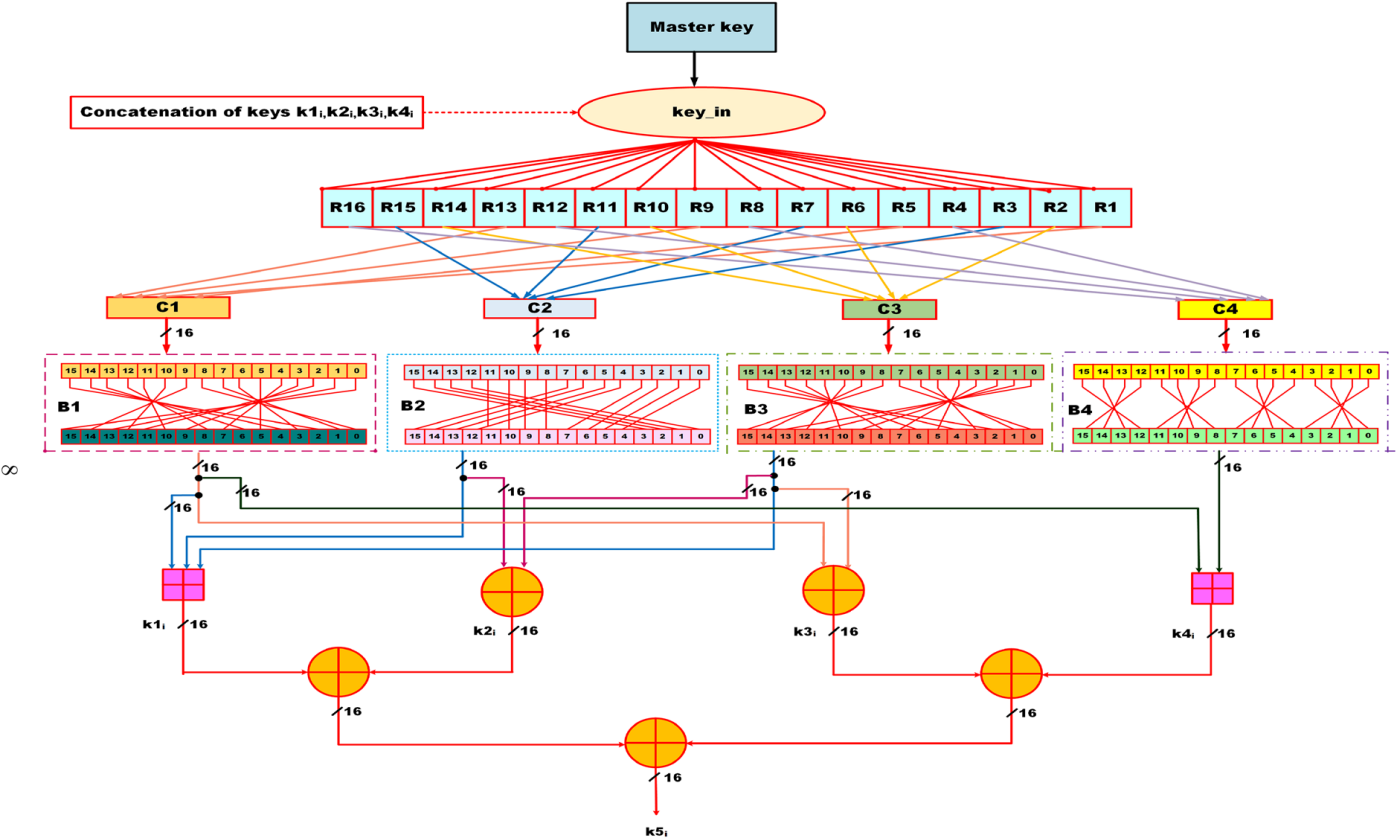
Novel key schedule mechanism of the proposed lightweight ARX cipher, illustrating subkeys generation and iterative round key generation.

This subsection outlines the novel key schedule mechanism designed for the proposed lightweight ARX cipher, as illustrated in Fig. 2. The key schedule plays a pivotal role in enhancing randomness and unpredictability, thereby contributing significantly to the cipher’s overall security. The key schedule begins with a 64-bit master key, denoted as $key\_in$, which serves as the initial seed.

The input master key $key\_in$ is divided into sixteen segments each of 4-bit wide and are labeled as *R*1, *R*2,... *R*16 from the LSB. Next, these segments are concatenated in a specific order to form four 16-bit distinct blocks denoted as *C*1, *C*2, *C*3, and *C*4 which introduce the diffusion among the subkeys. The grouping is based on a predefined mapping strategy that intentionally disperses key bits across different positions to promote diffusion. To further improve randomness and non-linearity, the output from each of these 16-bit blocks undergoes a customized bit-swap operation, labeled as *B*1, *B*2, *B*3, *B*4, respectively. Each bit-swap block operates in a different pattern to shuffle the inputs which in-turn improves the randomness and non-linearity.

Following the bit-swap operation, modular addition and bitwise XOR operations are applied to its outputs to generate five distinct subkeys in parallel, denoted as $k1_{i}$, $k2_{i}$, $k3_{i}$, $k4_{i}$ and $k5_{i}$. Here the subkey $ks_{i}$ refers to the $s^{th}$ stage of the $i^{th}$ encryption round. As the proposed cipher consists of five rounds, this subkey generation process is executed iteratively for each round, producing a fresh set of subkeys in parallel for every round.

To ensure round wise uniqueness and avoid key repetition, a key update mechanism is employed. This mechanism derives the next round’s key input by concatenation on the subkeys $k1_{i}$, $k2_{i}$, $k3_{i}$, $k4_{i}$ generated in the current round, thus maintaining inter round key dependency. By assigning a unique, non repeating subkey to each internal stage of every encryption round, the proposed key scheduling strategy enhances the cipher’s resistance against cryptanalytic techniques, notably linear and differential attacks. This design ensures high diffusion in the key space and contributes significantly to the cipher’s overall security.

#### Key schedule mechanism

The complete sequence of operations for the key schedule mechanism is outlined as follows:

Step 1: Initialize the 64-bit master key, denoted as $key\_in$ which serves as the input to the key schedule.

Step 2: Divide $key\_in$ into sixteen segments each 4-bit wide from LSB are labeled as *R*1, *R*2,... *R*16.

Step 3: Form four 16-bit intermediate blocks by concatenating the segments in a pre-defined order, i.e., $C1 = (R1 \parallel R5 \parallel R9 \parallel R13)$, $C2 = (R3 \parallel R7 \parallel R11 \parallel R15)$, $C3 = (R2 \parallel R6 \parallel R10 \parallel R14)$ and $C4 = (R4 \parallel R8 \parallel R12 \parallel R16)$.

Step 4: Apply bit-swap operations to each of the concatenated groups to further increase diffusion and randomness: *B*1 = bitswap (*C*1), *B*2 = bitswap (*C*2), *B*3 = bitswap (*C*3), and *B*4 = bitswap (*C*4).

Step 5: Generate the four intermediate subkeys using as $k1_{i}$ = ((*B*1 $\boxplus$ *B*2) $\boxplus$ *B*3), $k2_{i}$,=(*B*3 $\oplus$ *B*2), $k3_{i}$=($B1$ $\oplus$ *B*3), $k4_{i}$ =( *B*1 $\boxplus$ *B*4).

Step 6: Derive the fifth subkey $k5_{i}$ as a combined XOR of the previously generated subkeys. ($k5_{i}$ = $k1_{i}$ $\oplus$ $k2_{i}$ $\oplus$ $k3_{i}$ $\oplus$ $k4_{i}$)

Step 7: Concatenate the subkeys $k1_{i}$, $k2_{i}$, $k3_{i}$, $k4_{i}$ to form a new 64-bit key, which is feedback as $key\_in$ for the next round’s subkey generation.

$$key\_{in}^{(i+1)} = k1_i \parallel k2_i \parallel k3_i \parallel k4_i$$.

## Security analysis of the proposed ARX cipher

This section presents a comprehensive security evaluation of the proposed lightweight ARX cipher, focusing on its robustness against three critical aspects: randomness assessment using statistical tests, avalanche effect analysis, and resilience to conventional cryptanalytic techniques for block ciphers such as linear and differential cryptanalysis.

### Statistical analysis of the proposed key schedule mechanism

The performance of the proposed key schedule mechanism was evaluated for randomness using the NIST SP 800-22 statistical test suite, a widely accepted benchmark comprising 15 standard tests designed to detect non-randomness in binary sequences by identifying statistical patterns^46^. For this evaluation, the key schedule mechanism was used to produce a bitstream of length $10^{6}$ bits. The Table 2, demonstrate that all statistical tests yielded p-values $\ge$ 0.01, thereby satisfying the threshold for randomness as defined by NIST guidelines. This outcome confirms the statistical soundness of the generated subkeys sequence and reinforces the reliability of the key schedule in contributing to the overall strength of the cipher.

**Table 2:** NIST statistical test results.

Test methods	P-value	Results
Frequency	0.085587	Pass
Block Frequency	0.867692	Pass
Cumulative Sums (forward)	0.289667	Pass
Cumulative Sums (inverse)	0.171867	Pass
Runs	0.883171	Pass
Longest run	0.514124	Pass
Rank	0.574903	Pass
FFT	0.534146	Pass
Non-Overlapping Template	0.883171	Pass
Overlapping Template	0.834308	Pass
Universal	0.964295	Pass
Approximate Entropy	0.883171	Pass
Random Excursions	0.574903	Pass
Random Excursions Variant	0.816537	Pass
Serial	0.366918	Pass
Linear complexity	0.719747	Pass

### Avalanche effect

**Fig. 3:**
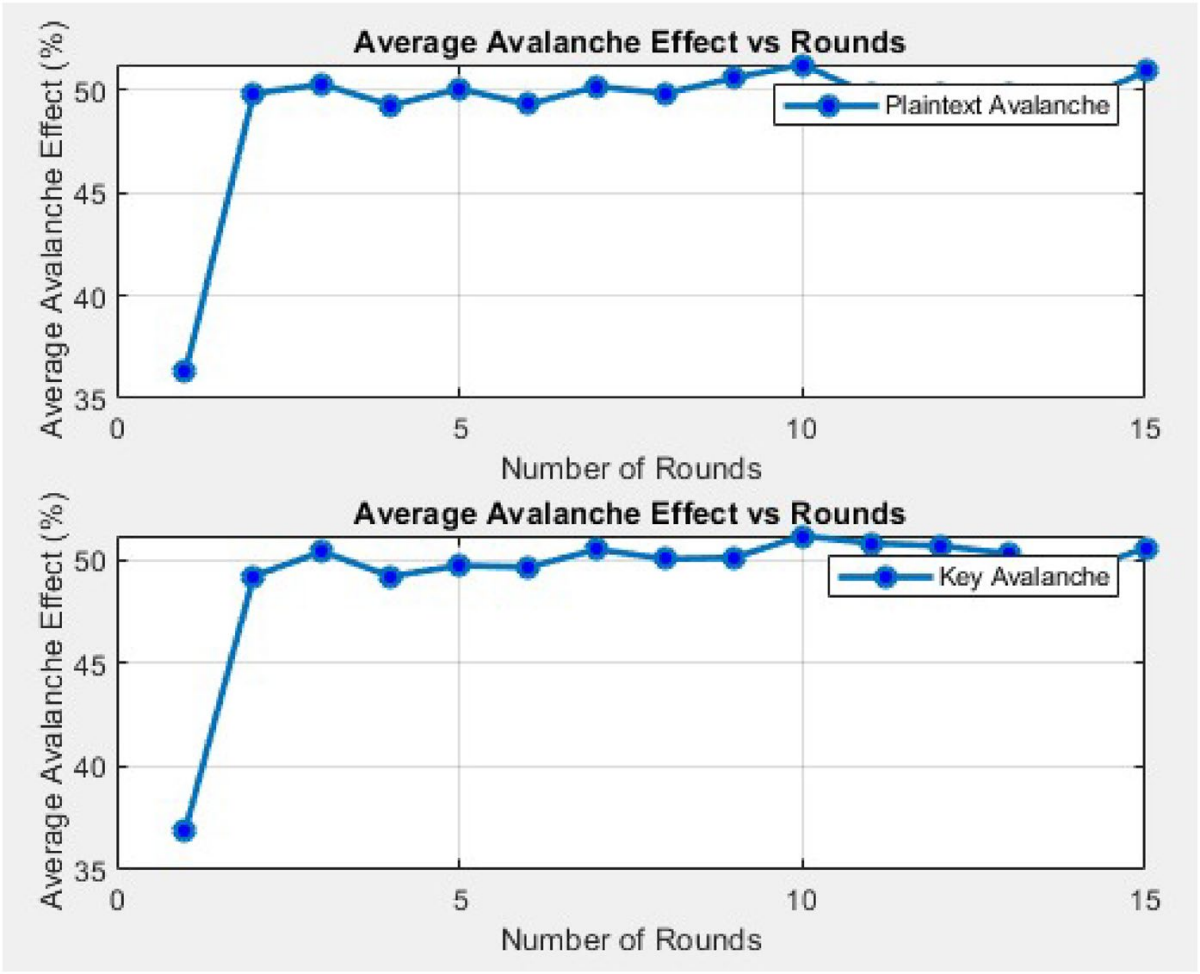
Average avalanche effect analysis showing the sensitivity of the proposed ARX cipher to single-bit changes in plaintext and key.

The avalanche effect is a critical property of secure cryptographic algorithms, ensuring that a minimal input change such as a single bit flip in either the plaintext or the key produces a widespread and unpredictable transformation in the output ciphertext. Mathematically, the avalanche effect is measured by calculating the ratio of the number of changed bits in the ciphertext due to a single bit flip in either the plaintext or the key to the number of bits in the ciphertext^47^.This diffusion property is crucial for resisting differential cryptanalysis, as it prevents the leakage of structural patterns from the input.To assess the avalanche characteristics of the proposed lightweight ARX cipher, a statistical experiment was conducted across 15 encryption rounds, using 1000 input samples per round. Two independent tests were performed: (1) Plaintext Avalanche Test – where one bit in the plaintext is flipped, and (2) Key Avalanche Test – where one bit in the encryption key is flipped. For each test, the average number of changed bits in the ciphertext was recorded and the results are expressed as a percentage of the 64-bit ciphertext as illustrated in Fig. 3. In the early rounds, such as Round 1, the average number of flipped bits was 22.18 bits (34.66%) for plaintext changes and 23.69 bits (37.02%) for key changes, indicating the onset of diffusion. By Round 3, the avalanche effect had stabilized near the ideal 50% mark 32.10 bits (50.16%) for plaintext and 31.96 bits (49.93%) for key changes. From Rounds 4 to 15, the avalanche effect remains stable and optimal with approximately 32 out of 64 ciphertext bits flipped on average in both tests. This suggests that by Round 5, the cipher reaches maximum diffusion, a critical marker for security.

### Linear cryptanalysis

Linear cryptanalysis is a form of known plaintext attack originally proposed by Matsui^48^, the attacker attempts to discover linear approximations that relate selected bits of the plaintext, ciphertext, and key with a probability significantly different from 0.5. To assess the resistance of the proposed ARX cipher against linear cryptanalysis, a full linear correlation bias analysis was conducted. This method involve by fixing a single bit (specifically the most significant bit) in randomly generated plaintexts and computing its correlation with each of the 64 bits in ciphertext. According to cryptanalytic standards used in the literature (e.g., AES, SPECK, PRESENT), a cipher is considered secure if the maximum observed bias is less than $2^{-8} \simeq 0.00391$ and it holds independent of the block size.

**Fig. 4:**
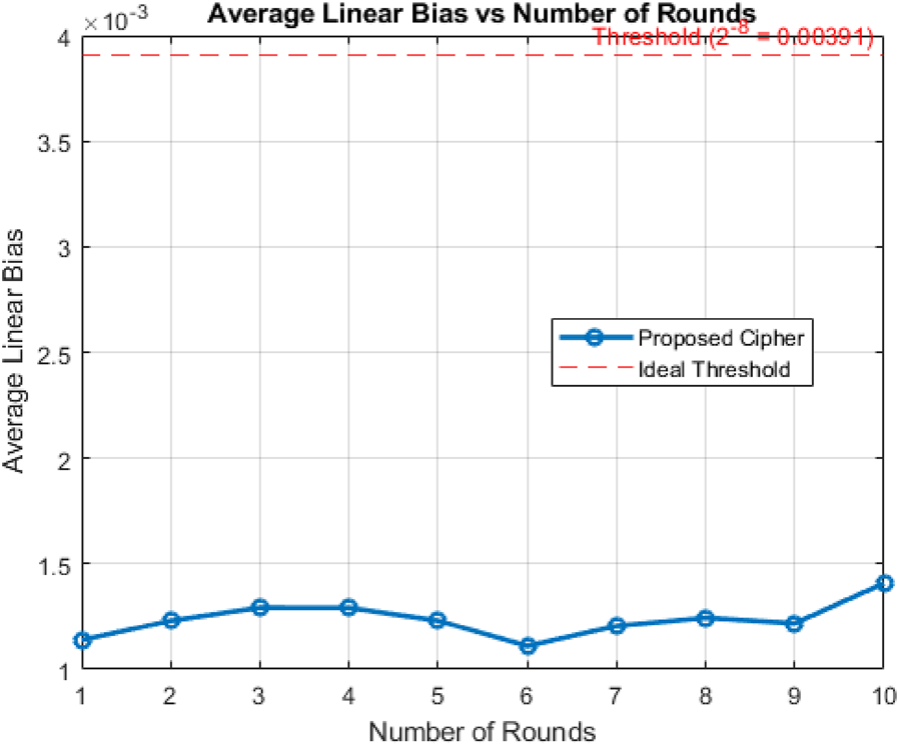
Linear cryptanalysis of the proposed lightweight ARX cipher showing the average linear bias across multiple rounds with a threshold reference of $2^{-8} = 0.00391$.

For each round, this analysis was iterated with $10^5$ random plaintexts to ensure statistical reliability. The absolute bias $\left| P - 0.5 \right|$ where *P* is the probability of bit agreement between the fixed plaintext bit and each ciphertext bit, was computed and averaged per round. The analysis was extended over 10 encryption rounds and it is observed that the measured average linear bias across all 64 bits in ciphertext remained well below the standard cryptanalytic threshold. Specifically, the average bias across multiple rounds ranged from 0.00110 to 0.001407, indicating no strong linear correlations exploitable by linear attacks. These results confirm that the proposed cipher achieves sufficient resistance against linear cryptanalysis as shown in the Fig. 4. Furthermore, since the bias remains well below the threshold even at 5 rounds, this indicates that five rounds of encryption are adequate from the perspective of linear attack resistance.

### Differential cryptanalysis

Differential cryptanalysis is a standard and powerful statistical technique for evaluating block cipher resistance by examining how differences in plaintext pairs affect the differences in the corresponding ciphertexts^49^. Specifically, it analyzes the propagation of an input difference $\Delta P = P_1 \oplus P_2$ through the cipher and the probability that a specific output difference $\Delta C = C_1 \oplus C_2$ results. The likelihood that a particular input difference leads to a given output difference is known as the differential probability (*DP*)^50^. A cipher is considered secure against differential attacks if all differential probabilities remain close to $2^{-n}$, where *n* is the cipher block size. For a 64-bit block cipher, the standard threshold is $2^{-32} \simeq 2.33 \times 10^{-10}$.

**Fig. 5:**
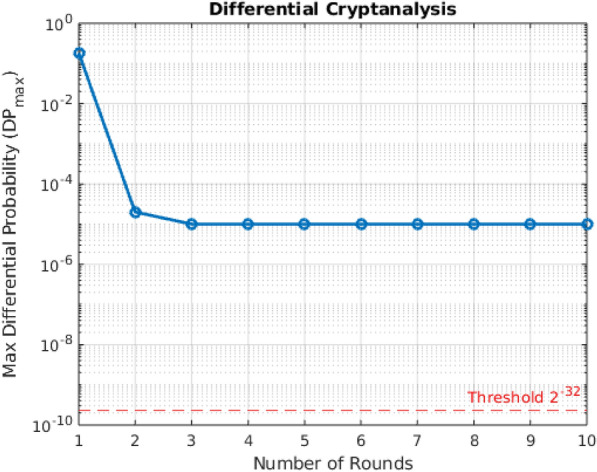
Differential cryptanalysis of the proposed lightweight ARX cipher showing the Maximum probability of differential characteristics across multiple rounds with a threshold reference of $2^{-32}$.

In this study, the differential analysis was conducted by encrypting $10^5$ randomly generated plaintext pairs with a fixed input difference $\Delta P$. The cipher was tested over 10 rounds of encryption and for each pair, the output difference was calculated and the most frequently occurring difference (i.e., the maximum differential probability $(DP_{max})$ was estimated for each round to identify any exploitable differential biases. The results of the differential cryptanalysis is illustrated in Fig. 5. Round 1 exhibited a relatively high maximum differential probability $(DP_{max})$ of 0.5476, indicating strong differential bias due to limited diffusion at the early stage. However, in Round 2, a significant improvement was observed, with $(DP_{max})$ sharply dropping to $2.00 \times 10^{-5}$ which is well below the theoretical threshold ($2^{-32} = 2.33 \times 10^{-10})$of for 64-bit block ciphers. From Rounds 3 through 6, $(DP_{max})$ values stabilized at $1.00 \times 10^{-5}$, reflecting near-ideal differential uniformity. These results confirm that the cipher achieves strong resistance to differential attacks within five rounds, and maintains consistent uniformity through subsequent rounds.

## Hardware analysis

**Fig. 6:**
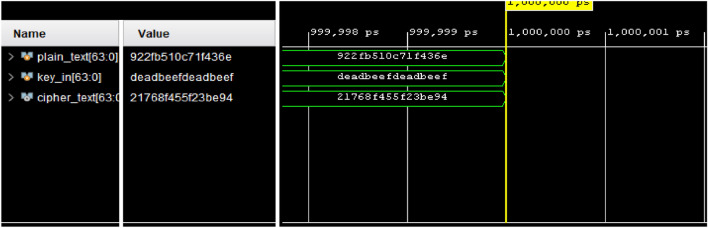
Simulation output verifying the functionality of the proposed lightweight ARX cipher encryption process.

**Fig. 7:**
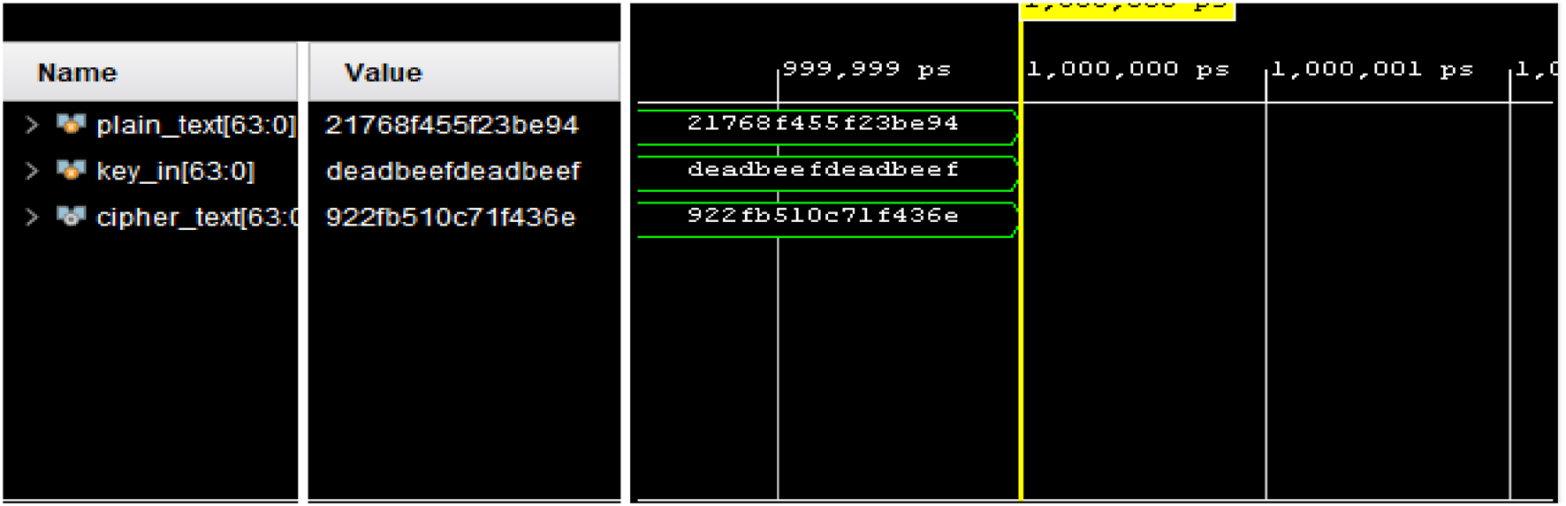
Simulation output verifying the functionality of the proposed lightweight ARX cipher decryption process.

This section presents the hardware implementation of the proposed ARX cipher on the Artix-7 (XC7A100T) FPGA. The hardware architecture is described using Verilog Hardware Description Language (HDL) and synthesized using the Xilinx Vivado Design Suite.The proposed lightweight ARX cipher encryption and decryption processes were functionally validated using predefined plaintext and key inputs. As shown in Figs. 6 and 7, the design was successfully simulated and verified, confirming the correctness of the hardware implementation for both operational modes.

**Fig. 8:**
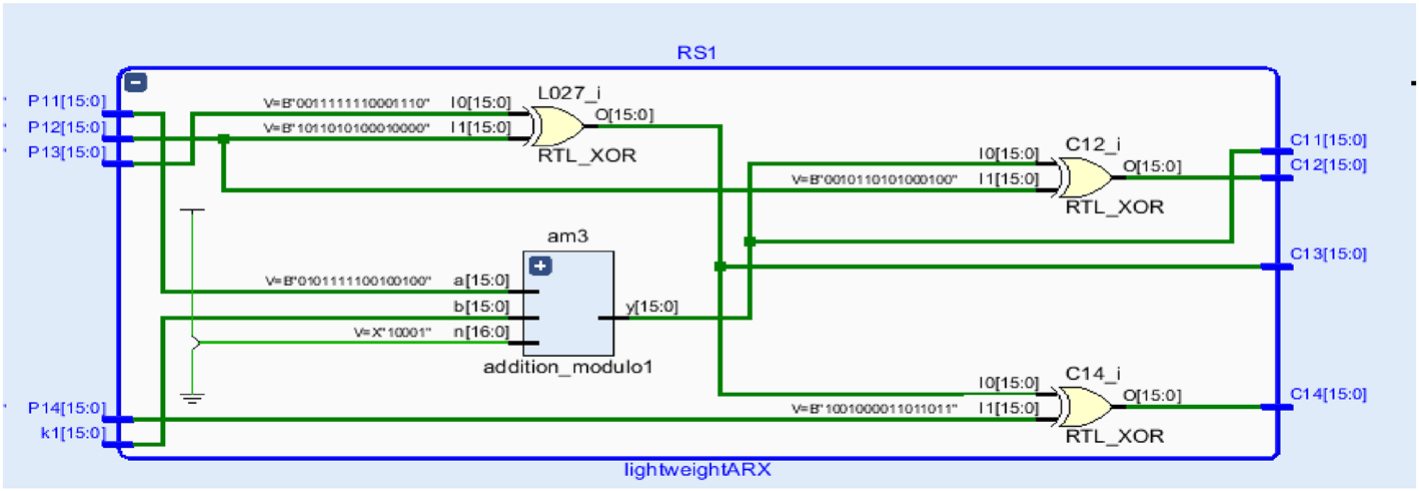
Register-transfer level (RTL) schematic for one stage of the ARX based encryption operation.

**Fig. 9:**
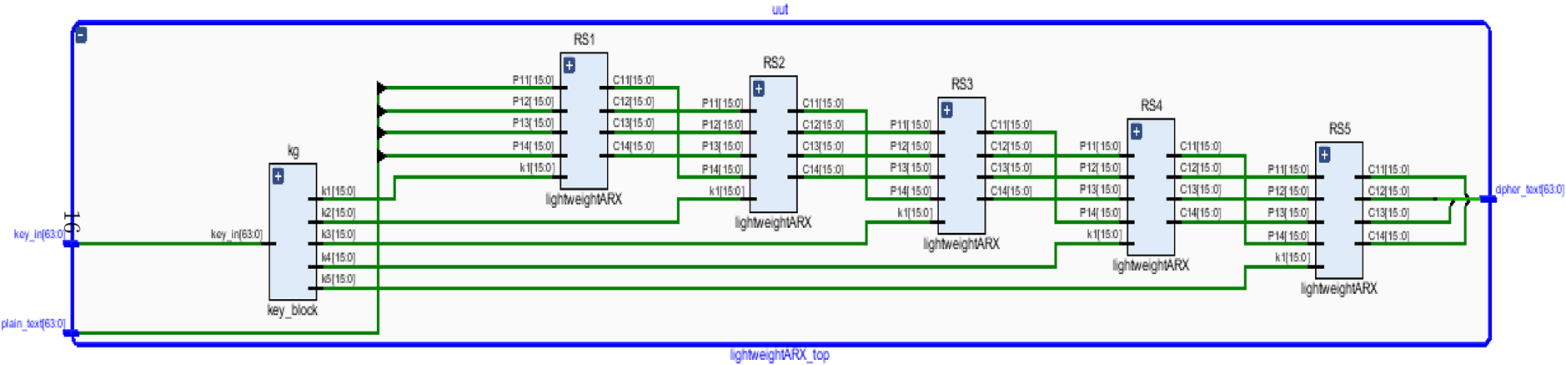
Register-transfer level (RTL) representation of the multi-stage encryption process implementing one round of the proposed ARX cipher.

The Register Transfer Level (RTL) view of the one stage of the ARX operation and the one round encryption process are illustrated in Figs. 8 and 9, which showcases the structural arrangement and datapath of the encryption logic.Based on the hardware implementation results presented in Table 3, a detailed comparative analysis shows the efficiency of the proposed lightweight ARX block cipher in terms of area utilization and power consumption. The proposed design implemented on the Artix-7 FPGA achieves the lowest area usage, requiring only 485 LUTs, which significantly reduces hardware when compared with other notable designs. For instance, it shows a 63.08% and 62.86% area reduction over the ARX^42^ and MRX^42^ ciphers respectively, both of which require over 1300 LUTs. Similarly, significant reductions are observed compared to SPECK (57.64%)^36^, Salsa20 (83.58%)^34^, and LEA (57.11%)^26^, proving the simpler architecture of the proposed cipher. Even when compared to the improved LEA cipher^38^, which is among the more optimized implementations, the proposed design still achieves a 9.34% area reduction.

**Table 3:** Hardware performance comparison of the proposed and existing ciphers on FPGA.

S. No.	Cipher	Device	Area (LUT)	Flipflops	Frequency (MHZ)	Power (W)
1	MBRISI^40^	Artix-7	492	0	23.66	0.042
2	ARX^42^	Artix-7	1314	–	–	40.458
3	MRX^42^	Artix-7	1306	–	–	40.115
4	SPECK^36^	PYNQ-Z2	1145	–	16.53	–
5	Salsa20^34^	Spartan-6	2955	–	48	–
6	Chacha20^32^	Virtex-7	940	–	161	–
7	LEA^26^	Virtex-5	1131	645	126.23	–
8	ILEA^38^	Virtex-6	535	–	270.85	–
9	Proposed	Artix-7	485	0	48.544	0.13

In terms of operating frequency and power, the proposed cipher maintains an excellent balance. It operates at 48.544 MHz, outperforming MBRISI (23.66 MHz) and SPECK (16.53 MHz), and closely matching Salsa20 (48 MHz), while consuming a low power of just 0.13 W. This makes it one of the most efficient implementations among those compared. Moreover, the design uses no flip-flops, indicating a fully combinational logic style, which contributes to lower dynamic power consumption and simplified timing closure. These results collectively demonstrate that the proposed cipher offers a highly optimized solution in terms of hardware, power, and speed, making it ideal for lightweight cryptographic applications in resource-constrained environments.

## Application analysis of proposed ARX block cipher

The proposed lightweight ARX-based block cipher is specifically designed for image encryption applications, addressing the growing demand for secure and efficient multimedia data protection. To evaluate its effectiveness, the cipher was applied to a variety of test images sourced from the USC-SIPI image database^51^. Statistical analyses included histogram uniformity, information entropy, pixel correlation, NPCR (Number of Pixels Change Rate), and UACI (Unified Average Changing Intensity), all of which confirmed the cipher’s ability to effectively obscure image structures. In addition, perceptual quality metrics such as Peak Signal-to-Noise Ratio (PSNR), Mean Squared Error (MSE), and Structural Similarity Index Measure (SSIM) were calculated to assess the visual distortion between the original and encrypted images.

### Visual perception

**Fig. 10:**
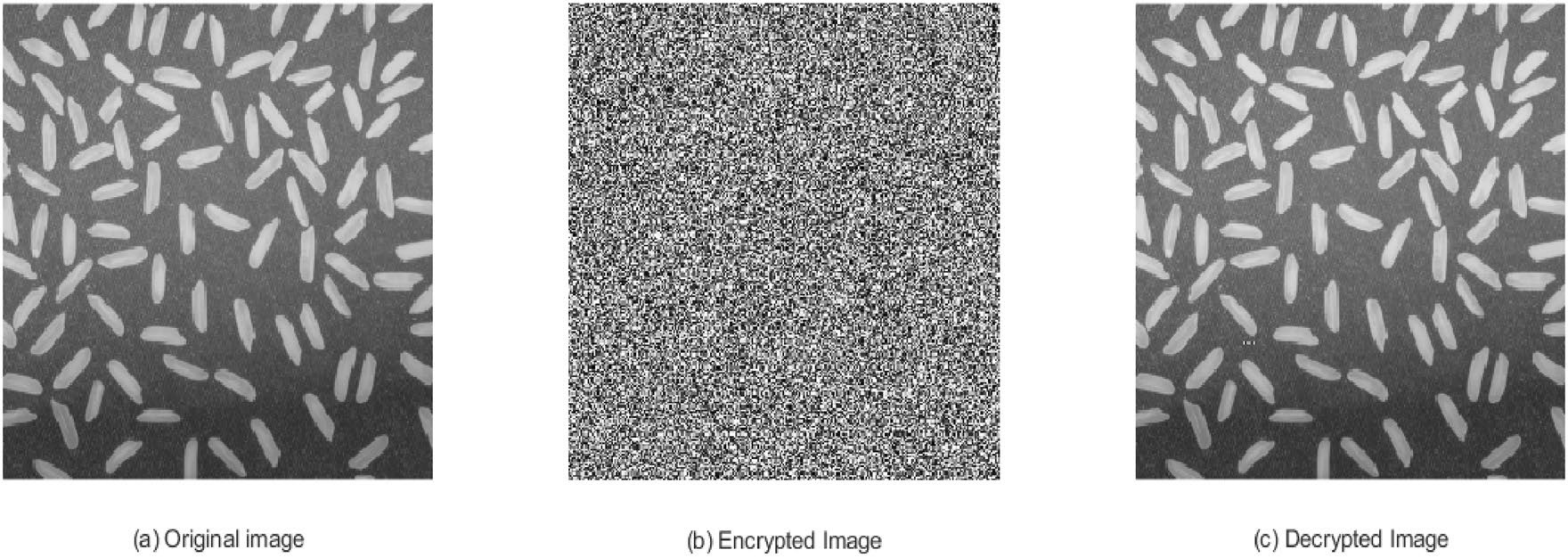
Rice image: (**a**) Original image, (**b**) Encrypted image, (**c**) Decrypted image.

**Fig. 11:**
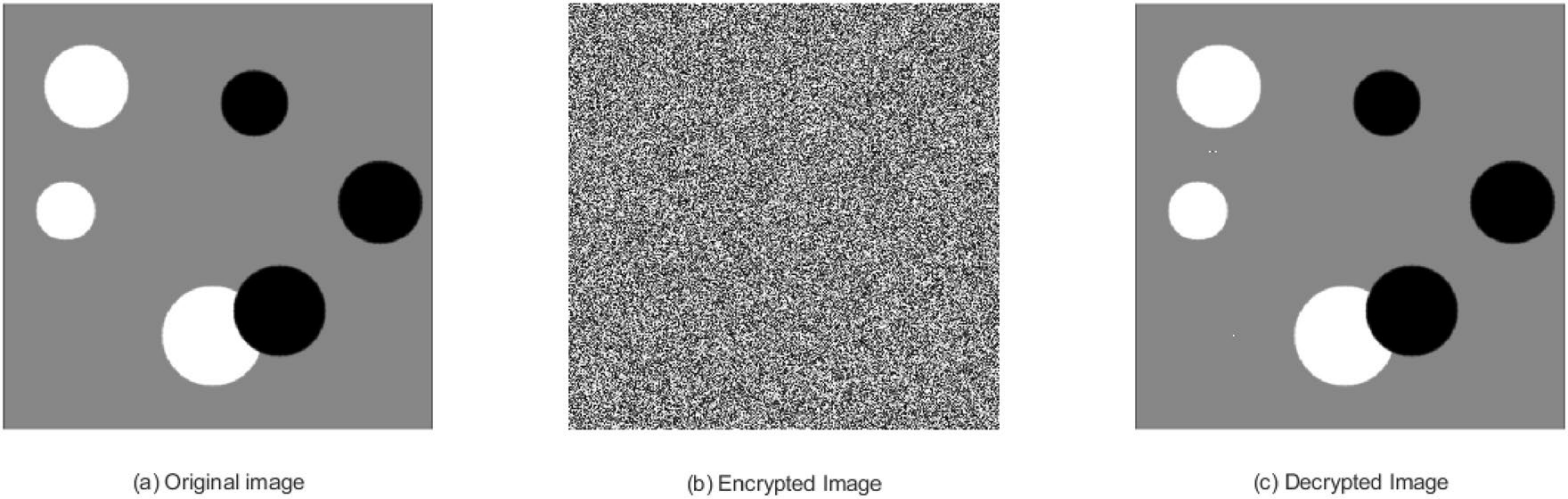
Coin image: (**a**) Original image, (**b**) Encrypted image, (**c**) Decrypted image.

To evaluate the visual quality and perceptual integrity of the encrypted and decrypted images generated by the proposed lightweight ARX block cipher implemented in MATLAB, two standard 256$\times$256 grayscale images are utilized, as shown in Figs. 10a and 11a. Each pixel in the original image is represented by an 8-bit binary value. During encryption, four consecutive pixel values are grouped to form a 64-bit plaintext block. The cipher processes each block sequentially, applying encryption using distinct round keys derived from the key schedule mechanism.

The resulting encrypted images, presented in Figs. 10b and 11b, display a highly randomized pixel distribution, effectively concealing the visual content of the original images. This visual randomness demonstrates the cipher’s ability to achieve strong confusion and diffusion properties. Furthermore, the decryption process accurately reconstructs the original images, as shown in Figs. 10c and 11c, thereby validating both the correctness and reversibility of the cipher.

### Histogram analysis

**Fig. 12:**
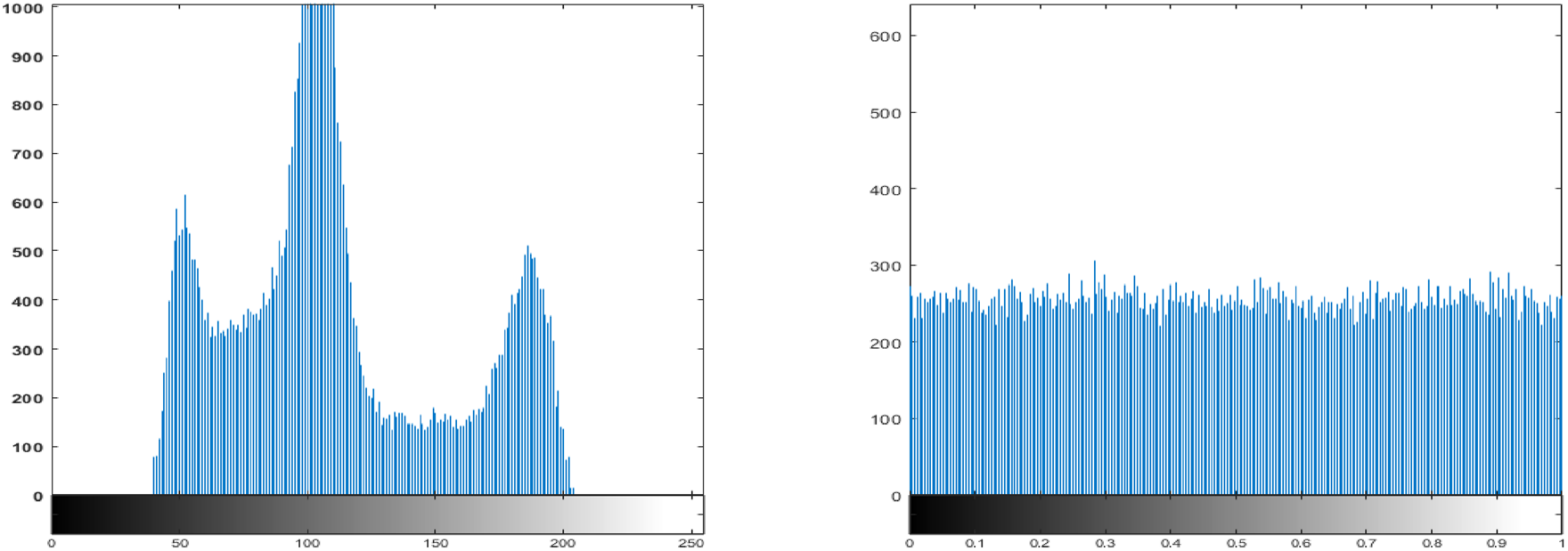
Histogram analysis of the Rice image: (**a**) Original image, and (**b**) Encrypted image using the proposed lightweight ARX cipher.

**Fig. 13:**
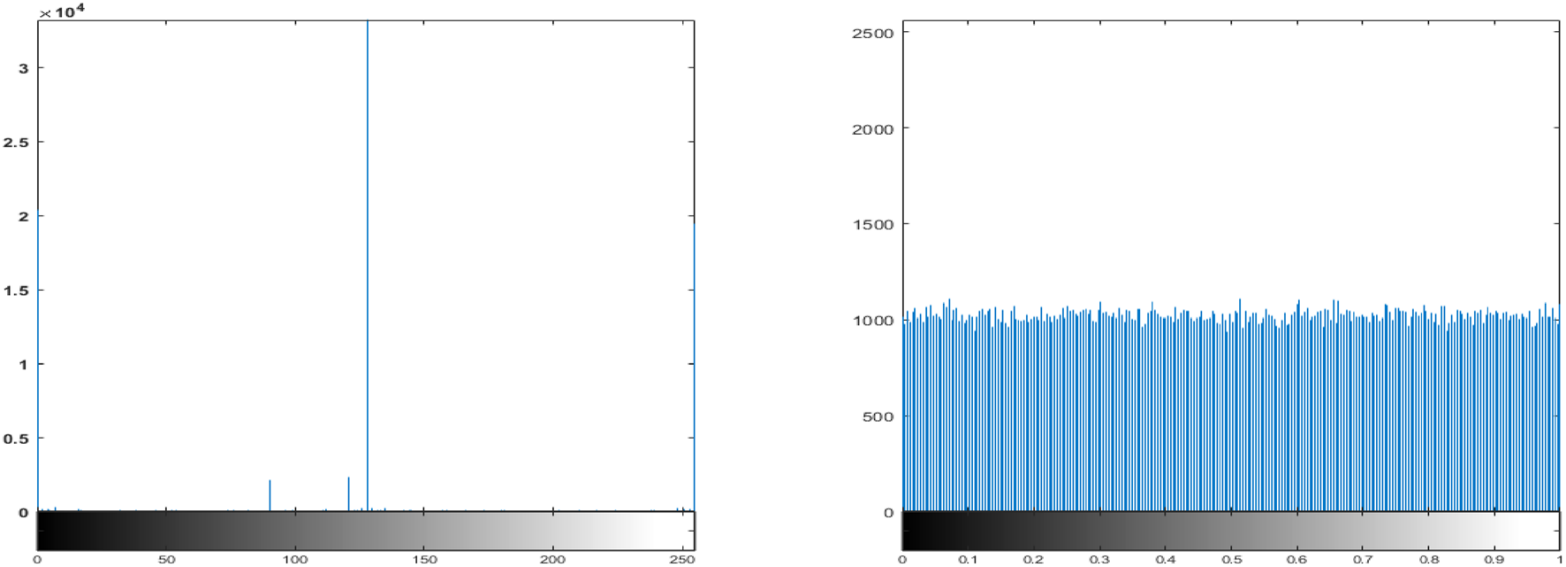
Histogram analysis of the coin image: (**a**) Original image, and (**b**) Encrypted image using the proposed lightweight ARX cipher.

Histogram analysis is a widely used technique to evaluate the statistical distribution of pixel intensities in an image and is computed using Eq. (1), as suggested in^52^. Figures 12a and 13a show the histograms of two original images, which exhibit characteristic non-uniform distributions corresponding to the visual content of each image. In contrast, the histograms of the encrypted images, shown in Figs. 12b and 13b, appear uniformly flat, indicating a random distribution of pixel intensities.This uniformity in the histograms of the encrypted images is a strong indication that the proposed lightweight ARX block cipher effectively conceals the original pixel information, thereby enhancing the encryption security by resisting statistical attacks.

1 $$\begin{aligned} \text {var}(X) = \frac{1}{m^2} \sum _{i=1}^{m} \sum _{j=1}^{m} \frac{(x_i - x_j)^2}{2} \end{aligned}$$

here, $x_{i}$ and $x_{j}$ denote the number of pixels with grayscale intensity value *i* and *j*, respectively, while *m* denotes the total number of possible grayscale levels, typically 256 for an 8-bit image.

### Entropy analysis

To evaluate the randomness introduced by the proposed encryption scheme, entropy analysis was performed on both original and encrypted images. As shown in Table 4, the encrypted images consistently exhibit entropy values close to 7.9 i.e., close to the theoretical maximum of 8 for an 8-bit grayscale image. This indicates that the pixel intensity distribution in the encrypted outputs is highly uniform, reflecting a strong degree of randomness and minimal predictability. The entropy values were computed using the standard formula given in Eq. (2), as outlined in^53^.

2 $$\begin{aligned} \text {Entropy} = \sum _{i=1}^{N} p(i) \log _{2} \left( \frac{1}{p(i)} \right) \end{aligned}$$

In this expression, p(*i*) denotes the probability of occurrence of the $i^{th}$ grayscale intensity level, and N is the total number of distinct intensity levels (typically 256 for 8-bit images). High entropy values confirm that the encryption process effectively masks the original image content, ensuring resistance against attacks.

**Table 4:** Entropy and correlation coefficient for various images.

Image	Entropy	Correlation coefficient
Rice	7.992	$-0.002$
Circle	7.993	$-0.0008$
Cameraman	7.997	0.0001
Coins	7.997	0.0001

### Correlation analysis

Correlation analysis is performed to measure the degree of similarity between adjacent pixel values in an image, serving as another key metric for evaluating the effectiveness of the encryption algorithm. The correlation coefficient ranges from $-1.0$ to 1.0, where a value of 1.0 indicates perfect positive correlation, and $-1.0$ indicates perfect negative correlation^54^.

**Fig. 14:**
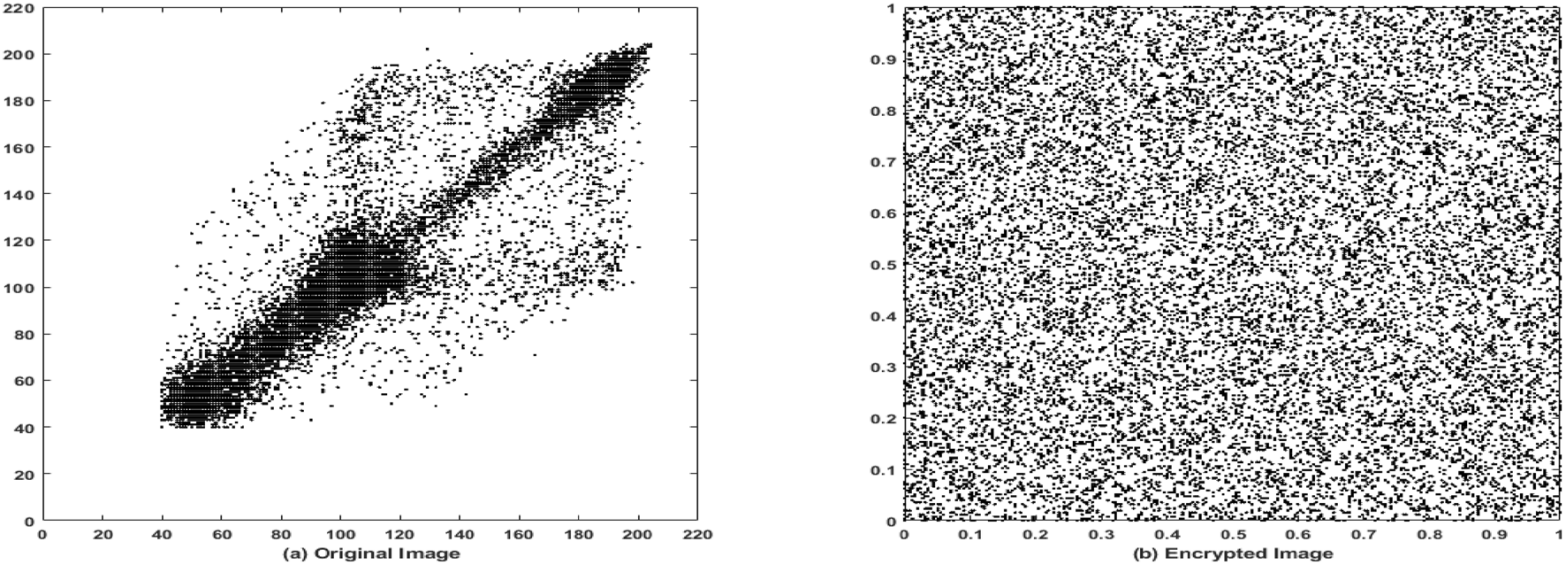
Correlation analysis of rice image. (**a**) Original image, (**b**) Encrypted image using the proposed lightweight ARX cipher.

**Fig. 15:**
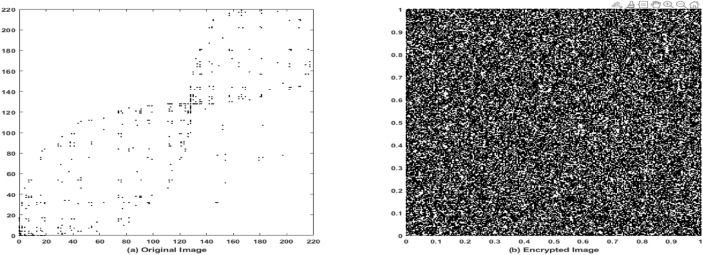
Correlation analysis of coin image. (**a**) Original image, (**b**) Encrypted image using the proposed lightweight ARX cipher.

In this study, the correlation coefficients of both the original and encrypted images have been computed using Eqs. (3)–(6) across various standard test images. The results are summarized in Table 4. As shown, the encrypted images consistently yield correlation values close to zero. Figures 14a and 15a illustrate the correlation distribution of adjacent pixels in the original images, which display strong directional clustering and high correlation. In contrast, Figs. 14b and 15b show the pixel correlation distribution after encryption, revealing a highly dispersed pattern. This reduction in correlation confirms that the proposed lightweight ARX block cipher introduces strong diffusion and effectively disrupts the inherent image structure.

3 $$\begin{aligned} C&= cov(s,t)/\sqrt{V(s)}\sqrt{V(y))} \end{aligned}$$

4 $$\begin{aligned} cov(s,t)&=M(s-M(s)) (t-M(t)) \end{aligned}$$

5 $$\begin{aligned} M(s)&=1/N\sum _{i=1}^{N}s_{i} \end{aligned}$$

6 $$\begin{aligned} V(s)&= 1/N\sum _{i=1}^{N}((s_{i})-M(s))^2 \end{aligned}$$

here, N denotes the total number of pixels used in the computation, M(s) and M(t) represent the mean values of the pixel sets *s* and *t*, respectively, and V(s), V(t) are the corresponding variances. The function cov (s,t) denotes the covariance between pixel pairs *s* and *t*, and *C* is the resulting correlation coefficient.

The results demonstrate that the encrypted images achieve high entropy and significantly reduced correlation coefficients approaching zero, which confirms the proposed cipher effectiveness in eliminating pixel-wise dependencies and ensuring strong statistical security.

### Peak Signal to Noise Ratio (PSNR) and Mean Square Error (MSE)

The Peak Signal-to-Noise Ratio (PSNR) quantifies the ratio between the maximum possible signal power and the noise power that affects the fidelity of an image^55^. It is widely used to assess the quality of reconstructed or decrypted images. Similarly, the Mean Squared Error (MSE) evaluates the average squared difference between corresponding pixel values in the original and encrypted images. These two metrics are inversely related i.e., higher MSE values result in lower PSNR, indicating poorer image quality.

In this study, both PSNR and MSE have been computed using Eqs. (7) and (8), and the results for various standard test images are presented in Table 5. The values confirm the effectiveness of the decryption process in preserving image quality.

7 $$\begin{aligned} PSNR = 10log_{10} \left( \frac{Max^{2}}{MSE} \right) \end{aligned}$$

where, *Max* denotes the maximum possible pixel value of the image, which is 255 for 8-bit grayscale images.

8 $$\begin{aligned} MSE= \frac{1}{MN}\sum _{i=1}^{M}\sum _{j=1}^{N}\left[ I(i,j) - E(i,j) \right] ^{2} \end{aligned}$$

here, M and N are the height and width of the image, respectively. I(i,j) and E (i,j) are the pixel values at the position (i,j) in the original and encrypted (or decrypted) images.

**Table 5:** Estimated values of PSNR, MSE, NPCR, UACI and SSIM for various images.

Image	PSNR (db)	MSE	NPCR (%)	UACI (%)	SSIM
Moon	10.12	6261.6	99.598	33.569	0.01
Rice	8.07	7552.3	99.592	33.28	0.01
Circle (bright & dark)	9.02	8134.0	99.61	31.63	0.01
Peppers	8.47	8395.4	99.56	33.52	0.01
Clock	6.9	12179.0	99.59	33.41	0.01
Airplane	6.68	10839.0	99.59	33.60	0.01
Aerial	9.15	7670.8	99.59	33.38	0.01

### Structural Similarity Index (SSIM)

The Structural Similarity Index (SSIM) is used to measure the structural difference between the original and encrypted images^56^. SSIM values range from 0 to 1, with 1 indicating identical images and 0 indicating no similarity. As shown in Table 5, the SSIM values between the original and encrypted images are very low, confirming that the encryption process effectively obscures structural information and enhances security.

### Number of Pixel Change Rate (NPCR) and Unified Average Changing Intensity (UACI)

Image security is strengthened when a slight change in the input image leads to a significantly different encrypted output^57^. To evaluate this sensitivity, two key metrics are used: Number of Pixel Change Rate (NPCR) and Unified Average Changing Intensity (UACI). Let $Z_{a}$ and $Z_{b}$ be two cipher images differing by one pixel in their input.For pixel coordinates $Z_{1}$(i,j) and $Z_{2}$(i,j), the binary difference term *B*(*i*, *j*) is defined in Eqs. (9) and (10).

9 $$\begin{aligned} B(i,j)&=0 if (Z_{a}(i,j) = Z_{b}(i,j)) \end{aligned}$$

10 $$\begin{aligned} B(i,j)&=1 if (Z_{a}(i,j) \ne Z_{b}(i,j)) \end{aligned}$$

Equation (11) can be used to express the NPCR as

11 $$\begin{aligned} NPCR = \sum _{i,j} B(i,j)/T \times (100 \%) \end{aligned}$$

Equation (12) defines the UACI as

12 $$\begin{aligned} UACI = \sum _{i,j} |Z_{1}(i,j) - Z_{2}(i,j)|/T_{p} \times Max \times (100 \%) \end{aligned}$$

here, $T_{p}$ is the total number of pixels and *Max* is the maximum pixel value (255 for 8-bit images). Table 5 presents the computed NPCR and UACI values using Eqs. 11 and 12, confirming high sensitivity and strong security when values approach ideal thresholds.

## Conclusion

This paper introduced a novel lightweight ARX-based block cipher specifically designed for secure image encryption applications. The proposed design features a custom key schedule scheme with multiple subkeys and a multi-stage internal structure within each round to ensure robust diffusion and confusion. A comprehensive evaluation was conducted to assess both the cryptographic strength and application performance of the cipher. Statistical randomness was verified using the NIST SP 800-22 test suite, confirming that all statistical tests yielded p-values $\ge 0.01$. The avalanche effect analysis demonstrated that small changes in plaintext or key result in approximately 50% bit changes in ciphertext, validating strong sensitivity and diffusion close to ideal behavior. Linear and differential cryptanalysis showed low exploitable biases and well below standard thresholds, indicating strong resistance to these classical attacks with five encryption rounds. For application-specific evaluation, the cipher was tested on standard images. The encrypted images exhibited high perceptual distortion, supported by metrics such as PSNR, SSIM, and MSE. Additionally, low correlation coefficients and uniform histograms validated the cipher’s effectiveness in concealing visual data.

Given its security strength, lightweight complexity, and suitability for image-based applications, altogether the proposed cipher represents a promising candidate for secure multimedia encryption in constrained environments.

## Data Availability

The data used and/or analyzed during this study are available from the corresponding author upon reasonable request.

## Code availability

All the relevant code used to generate the results in this paper and supplementary information is available from the corresponding author upon reasonable request.
